# Healthcare access, symptom burden, and psychological impact in hypertrophic cardiomyopathy: a multinational patient-driven survey

**DOI:** 10.1016/j.ijcrp.2025.200485

**Published:** 2025-08-04

**Authors:** Emil Tsenov, Jolanda Van der Velden, Matteo Pinciroli, Maurizio Pieroni, Franco Cecchi, Iacopo Olivotto, Niccolò Maurizi

**Affiliations:** a European HCM Patient Foundation, Vienna, Austria; b Department of Physiology, Amsterdam UMC, Vrije Universiteit Amsterdam, Amsterdam, the Netherlands; c Amsterdam Cardiovascular Sciences, Heart Failure & Arrhythmias, Amsterdam, the Netherlands; d AICARM Italian Association for Cardiomyopathies, Florence, Italy; e Cardiomyopathy Unit, Careggi University Hospital, Florence, Italy; f Meyer Children's Hospital IRCCS, Florence, Italy; g Service of Cardiology, University Hospital of Lausanne (CHUV), Lausanne, Switzerland

**Keywords:** Hypertrophic cardiomyopathy, Quality of life, Patient centered outcomes

## Abstract

**Background and aims:**

Hypertrophic cardiomyopathy (HCM) is a complex genetic heart disease with significant clinical, psychological, and socioeconomic implications. While research has focused on pathophysiology and treatment, patient-reported experiences remain underexplored.

**Methods:**

A cross-sectional, multinational online survey was distributed between December 2024 and February 2025, targeting individuals diagnosed with HCM in Europe. The questionnaire included sections on demographics, symptom burden, impact on daily life, medical management, and psychological well-being. Data were analyzed descriptively, with subgroup analyses based on geography, employment, and healthcare access.

**Results:**

A total of 337 qualifying participants from 18 European countries completed the survey. They were mainly diagnosed because of symptoms (107, 42 %). Specifically, shortness of breath and fatigue had an overall high impact on quality of life, both at diagnosis and at the time of survey (3.09/5 vs 2.93/5; 3.23/5 vs 3.46/5, respectively). With HCM diagnosis, the proportion of patients engaged in low to moderate activities increased significantly (87 % vs 50 %, p < 0.01) and one major psychological complaint was weight gain (71, 49 %). Twenty-two (15 %) patients reported having lost their job because of HCM; 46 (14 %) reported a limitation in working hours as well as limitation in the kind of work performed (32, 9 %), due to the disease. Despite a significant psychological burden access to mental health support was limited, as only 15 % of patients regularly consulted a psychologist.

**Conclusions:**

This survey highlights critical gaps in HCM management, including healthcare accessibility, persistent symptom burden, and unmet psychological needs. Improved care pathways, mental health integration, and workplace accommodations are essential to enhance patient-centered HCM management across Europe.

## Graphical abstract

**Figure:**
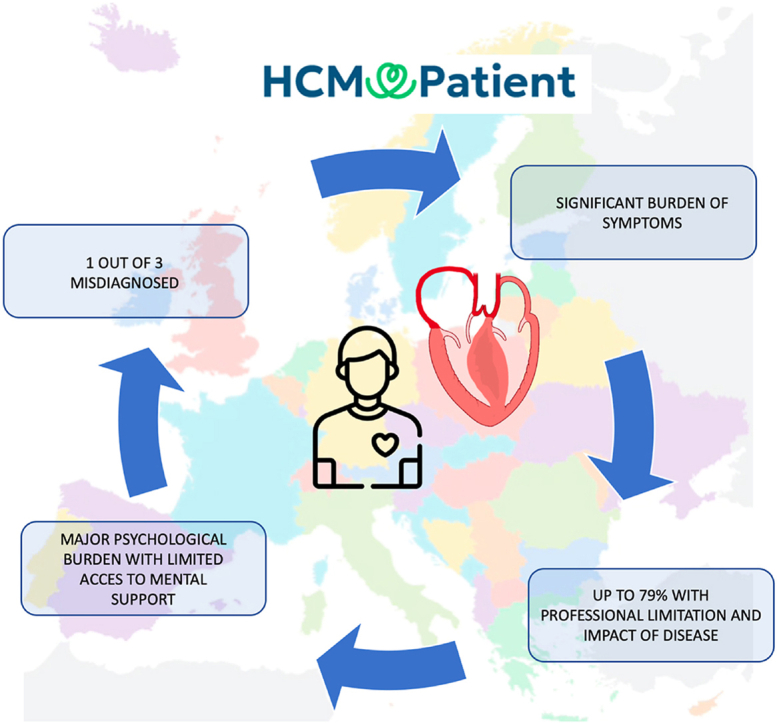

What is known?

Hypertrophic cardiomyopathy (HCM) is the most prevalent inherited cardiac disease, known for its clinical heterogeneity and significant impact on quality of life. Despite increasing emphasis on patient-centered care, little is known about the lived experience of individuals with HCM, including their access to specialized care, the burden of symptoms over time, consequences on work and daily activities, and psychological consequences. Moreover, prior studies on quality of life in HCM have often been small-scale, clinician-driven, and lacking international scope, leaving gaps in understanding how healthcare systems and social determinants influence outcomes across different populations.

What this study adds on?

This study presents the first large-scale, patient-initiated, multinational survey capturing the real-world experiences of individuals with HCM across 18 European countries. By collecting data from 337 patients, the survey sheds light on underrecognized aspects of HCM, including diagnostic delays, persistent symptom burden despite treatment, reduction in physical activity, and significant emotional distress. Importantly, the study emphasizes the lack of structured psychological support, despite widespread mental health concerns, and underscores the need for more integrated care models. This work contributes unique patient-generated evidence to inform policy changes, guide research priorities, and support a more holistic and equitable approach to 10.13039/501100014603HCM care in Europe.

Hypertrophic cardiomyopathy (HCM) is the most prevalent inherited cardiac disorder, affecting approximately 1 in 350 individuals worldwide [1,2]. Despite advances in diagnosis, management, and treatment, HCM remains a complex and heterogenous disease, significantly impacting patients' quality of life, mental health, and socioeconomic stability [3]. While extensive research has focused on genetics, clinical manifestations, and therapeutic strategies, relatively little attention has been given to the real-world experiences and unmet needs of patients living with HCM [[4], [5], [6], [7]]. HCM can present many challenges for patients and families, including morbidity, fears about disease-related complications, implications for daily activities and concerns about inheritance. Patients and caretakers should be therefore empowered in shared-decision making regarding relevant healthcare policies, services, technologies and research priorities [7]. However, despite widely advocated, no specific patients-initiated investigations on quality of life or disease burden has been performed in patients with HCM. The Kansas City Cardiomyopathy Questionnaire (KCCQ), the gold standard tool used in trials to assess the quality of life of patients with heart failure [8], has never been validated against hard endpoints in genetic cardiomyopathies [8]. As an example, the only validation study assessing KCCQ in obstructive HCM performed a qualitative cognitive debriefing to evaluate with patient interviews whether the questionnaire was understandable and pertinent to patients with obstructive HCM [8]. To bridge this knowledge gap, the European HCM Patient Foundation, a non-profit association of patients with HCM at European level, in coordination with national local patient organization, conducted the HCM Patient Survey 2025. It's a large-scale patient-initiated investigation aimed at capturing the perspectives, disease related challenges, and expectations of patients diagnosed with HCM across Europe. By systematically collecting data from spontaneously auto-enrolled patients in 18 European countries, this initiative provides valuable insights into the lived experience of HCM patients, beyond clinical assessments.

## 1 Methods

### 1.1 European HCM Patient Foundation and survey creation

The online survey among HCM patients in the European Union and the EFTA area (Switzerland, Norway, Iceland and Liechtenstein) is a cross-sectional study that was organized independently by the ‘HCM Patient Foundation’, which is a non-profit organization aimed at uniting and support patients with HCM located in the European Union. Among the objectives of the Foundation are working with the local and European authorities to improve the healthcare of HCM patients and protect their interests as well as helping to raise public awareness about HCM and its associated comorbidities.

The present survey was elaborated entirely by patients associated with the Foundation and led by ET, himself a patient affected by HCM. The questionnaire, developed specifically for this study, was composed of 97 questions, with multiple choice pre-defined answer with the possibility to add free text. For its creation a focus group within the patient council of the HCM Patient Organization was established that used available scientific articles in the field. The questionnaire draft was internally tested within this group and subsequently reviewed and approved by members of the HCM Patient Foundation Scientific Council (N.M., I.O., F.C., J.V.D.V.).

Domains explored by the questions were: demographic, social and geographical characteristics of the participants (14 items), education and professional background, disease-related data, financial, psychological and lifestyle impact (20 items), diagnosis pathways and clinical metrics (41 items), interactions with the health system and proactiveness of patients (13 items), medications usage and main communication channels used to obtain HCM information (5 items). None of the questions were compulsory. The survey was performed via the Qualtrics survey management system and followed GDPR requirements. It has been available online and collected replies (snowballing via patients networks) between December 5th, 2024 and February 16th, 2025.

The countries were selected based on the area which the HCM Patient Foundation aims to cover (the European Union plus the EFTA countries). In order to ensure maximum coverage the survey was made available in 12 different languages - English, Bulgarian, Dutch, German, Greek, Italian, Polish, Portuguese, Romanian, Russian, Spanish, and Swedish. Every effort was made to reach patients in all European Union and EFTA countries but certain disbalance was inevitable as the level of coverage of these countries by patient organization is uneven and the level of cooperation varied significantly**.**

### 1.2 Inclusion criteria were

- Self-declared diagnosis of Hypertrophic Cardiomyopathy;
- Living permanently (more than 6 months per year) in the European Union and the EFTA area (Switzerland, Norway, Iceland and Liechtenstein);
- Accepting the general terms of use and complete anonymization of the data.

### 1.3 Survey analysis

Survey data analysis was performed by a member of the patient's committee (ET) and a member of the Scientific Board (NM). Continuous variables, reported as means with standard deviations or as medians with interquartile ranges for non-normal distributions, were compared between groups with Student's T-test or non-parametric tests, as appropriate. Categorical variables, reported as counts and percentages, were compared between groups with chi-square or Fisher exact tests. Variables with missing data were not included in the analysis.

A 2-sided P-value less than 0.05 was considered statistically significant. All analyses were performed using SPSS Statistics for Macintosh version 25.0 (IBM).

## 2 Results

### 2.1 Demographic and geographical characteristics of the participants

**Fig. 1:**
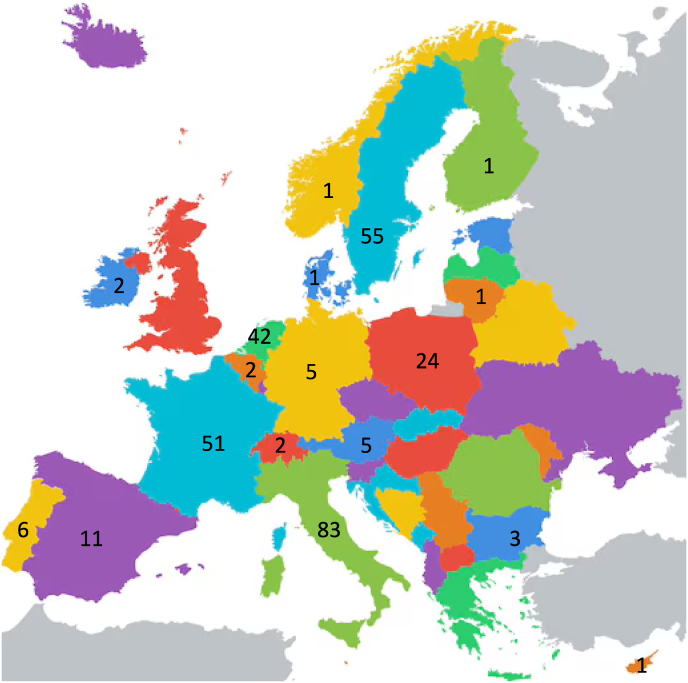
Geographic distribution of patients partecipating to the questionnaire across Europe.

The European HCM Patient Survey 2025 gathered responses from 337 eligible participants, selected from an initial pool of 424 respondents. A total of 87 (21 %) participants were excluded, mainly because they were non-EU citizens, individuals diagnosed with other cardiomyopathies, or did not agree with the study's terms and conditions (Fig. 1). The age distribution of the participants (276 respondents) showed a wide range, with 33/276 (12 %) younger than 30 years, 184/276 (67 %) between 30 and 60 years, and 59/276 (21 %) older than 60 years. The mean age at diagnosis was 38 years (185 respondents), though with significant variability: 66 (36 %) of respondents were diagnosed in childhood or adolescence (<30 years), 104 (56 %) between 30 and 60 years, and 15 (8 %) at or after 60 years.

The participants represented 18 European Union (EU) countries, with the highest number of responses coming from Italy (83, 24 %), Sweden (55, 16 %), France (51, 15 %) and the Netherlands (42, 13 %) (Fig. 1). Other countries, including Germany, Spain, and Poland, also contributed a substantial number of responses, while smaller patient groups were recorded from Eastern and Central European nations.

The survey also assessed the living environment of respondents, categorizing them based on whether they resided in large cities, small towns, or rural villages, as well as their proximity to specialized HCM care centers. Among participants, 35 % (95 % CI 31–38) lived in the capital of the country or in towns bigger than 100 000 people, 41 % (95 % CI 35–48) in small to medium-sized towns, and 24 % (95 % CI 19–27) in rural villages or remote areas. The distance from specialized cardiology centers varied considerably, with 26 % of respondents living within a considerable distance (from 2 h to a full day) from the nearest specialized facility, with a median time to travel of 2.3 [1; 3.4] hours. Notably, patients in rural areas were more likely to experience difficulties in accessing specialized care (p < 0.01), with 28 % (95 % CI 25–32) of those living in villages or remote locations reporting long travel times and logistical challenges in attending regular follow-ups.

Marital status and family planning decisions were also explored. Among respondents, 77 % (95 % CI 73–82) were married or in a long-term partnership, 17 % (95 % CI 14–23) were single, and 6 % (95 % CI 4–8) were divorced, widowed, or separated. Regarding parenthood, 66 % of participants had children, while 34 % (95 % CI 29–37) did not. Notably, 41 % (95 % CI 38–45) of respondents reported that their HCM diagnosis influenced their decision to have children. Additionally, 71 % (95 % CI 69–74) of those who had children opted for genetic testing in their offspring.

### 2.2 Education and professional characteristics

The survey revealed that the vast majority of patients (255, 96 %, 95 % CI 95–98) had completed secondary or higher education, and 39 % (95 % CI 36–42) held a university degree or higher. Despite this high level of educational attainment, many participants reported significant challenges in their professional life due to HCM. The investigation revealed further revealed significant occupational disparities by gender, with more significant consequences on the career of women versus that of men (31 % 95 % CI 27–35 vs. 21 % 95 % CI, 18–26, p < 0.05). Among the 100 respondents, all reported HCM as having an impact on their professional life. A total of 25 individuals (25 %, 95 % CI 17–34) described a minor limitation, typically involving limited adjustments at work without significant disruption. Twenty-three respondents (23 %, 95 % CI 15–31) reported a moderate impact, such as reduced ability to perform physically demanding tasks or requiring periodic absences. The majority - 52 individuals (52 %, 95 % CI 42–62) - reported a significant burden, including cases where patients had to reduce working hours, change occupations, or discontinue employment altogether.

### 2.3 Perception of disease burden

**Fig. 2:**
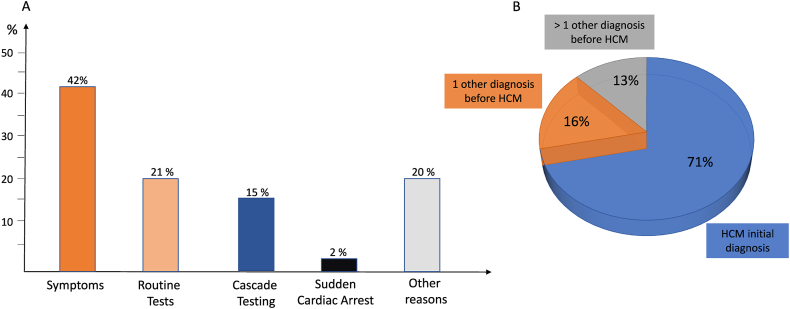
Self reported diagnostic pathways leading to the identification of Hypertrophic Cardiomyopathy Panel A represents an histogram depicting pathways leading to Hypertrophic Cardiomyopathy diagnosis. Panel B shows the percentage of the initial correct or delayed diagnosis.

Participants were mainly diagnosed because of symptoms (107, 42 %, 95 % CI 36–54) (Fig. 2). Among them, shortness of breath was the most prevalent (45, 42 %, 95 % CI 34–54), followed by fatigue (43, 40 %, 95 % CI 35–55), palpitations (42, 39 %, 95 % CI 32–41) and chest pain (41, 38 %, 95 % CI 32–44). Notably, 71 % (95 % CI 65–76) of participants received a correct initial diagnosis of HCM, while 29 % (95 % CI 23–34) experienced at least one prior misdiagnosis, leading to delays in appropriate disease management. Among misdiagnosed cases, 16 % (95 % CI 12–22) had received a single incorrect diagnosis, while 13 % (95 % CI 8–18) had been given multiple alternative explanations before HCM was recognized (Fig. 2).

**Fig. 3:**
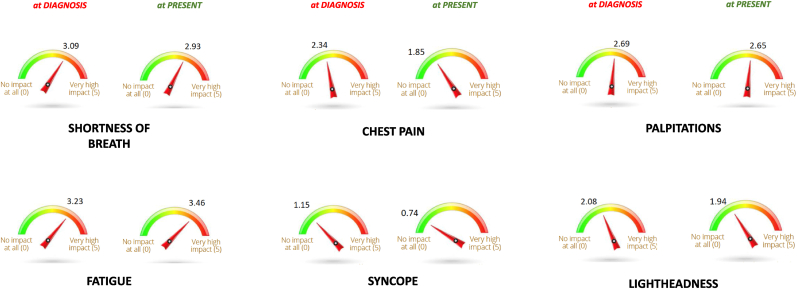
Evolution of perceived symptoms burden from diagnosis to present.

Over time, symptom burden evolved, with 77 % (95 % CI 65–82) of participants continuing to experience persistent symptoms despite treatment and lifestyle modifications. Specifically, shortness of breath and fatigue had an overall high impact on quality of life, both at diagnosis and at the time of survey (3.09/5 vs 2.93/5; 3.23/5 vs 3.46/5, respectively). On the contrary, the burden of chest pain and the occurrence of syncope, decreased after medical attention (2.34/5 vs 1.85/5; 1.15/5 and 0.74/5, p < 0.01, respectively) (Fig. 3).

**Fig. 4:**
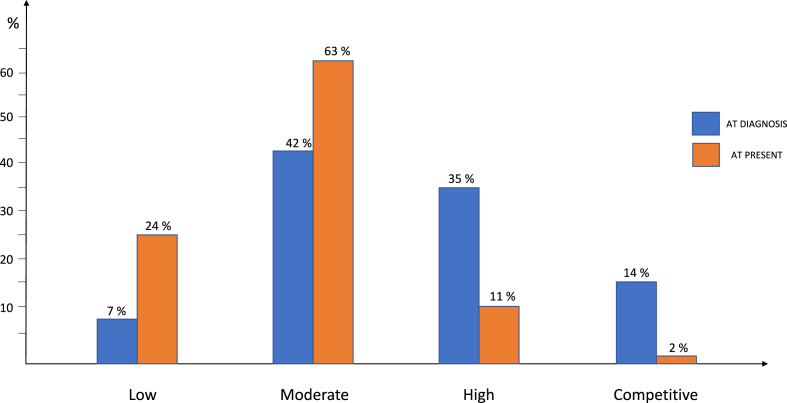
Level of engagement in physical activity at diagnosis and at present across the cohort.

The impact of HCM on physical activity was pronounced. At the time of diagnosis, 33 (14 %) of respondents engaged in competitive sports, 82 (35 %) participated in regular structured exercise, and 98 (42 %) engaged in light recreational activity (Fig. 4). However, after diagnosis, 63 % of respondents indicated that they had reduced their physical activity levels.

Specifically, only 4 (2 %) continued competing in sports, while the proportion of patients engaged in low to moderate activities increased significantly (87 % vs 49 %, p < 0.01) (Fig. 4).

### 2.4 Perception of disease care

A total of 84 (42 %, 95 % CI 38–48), 199 respondents) participants self-declared with obstructive HCM, and 233 (69 %, 95 % CI 61–73) were aware of their last ejection fraction and Nt-pro BNP values. The most frequently prescribed medications included beta-blockers (152, 86 % 95 % CI 82–93), calcium channel blockers (43, 24 % 95 % CI 20–29), and disopyramide (25, 14 % 95 % CI 10–19). A total of 173 (51.5 % 95 % CI 45–56) patients had experienced at least one change in medication since their initial diagnosis, with 64 (37.4 % 95 % CI 31–43) starting new drug therapies and (67, 20.5 % 95 % CI 15–26) discontinuing previous treatments. Beta-blockers were the medication with most commonly reported side effects and more impacting the quality of life (128, 40 % 95 % CI 37–45, 1.63/3), followed by calcium channel blockers (51, 15 %, 95 % CI 9–21, 1.33/3). Myosin inhibitors had side effects in 11 (3 % 95 % CI 1–5) of patients, with a perceived burden of 1.36/3 (Table 1).

**Table 1:** Self reported side effects of most common drugs prescribed in patients with Hypertrophic Cardiomyopathy.

Medication	Number of patients who experience side effects (on total cohort)	Average Weight of side effect (0–3)
Beta Blockers (Atenolol, Metoprolol, Bisoprolol, Nadolol)	128 (40 %)	1.63/3
Calcium-channel blocker (Amlodipine, diltiazem)	51 (15 %)	1.33/3
Disopyramide	22 (7 %)	1.32/3
Anticoagulants (Warfarin, Apixaban, heparin)	32 (9 %)	1.28/3
Myosin Inhibitor (Mavacamten)	11 (3 %)	1.36/3

Table 2 shows the trend of device implantation among countries and surgical procedures.

**Table 2:** Device implantation and surgical procedures trend per country. *Abbreviations: ICD: implantable cardioverter defibrillators, LVAD: Left Ventricular Assist Device; AfiB: Atrial Fibrillation; HCM: Hypertrophic Cardiomyopathies*.

	Total (n = 178)	Italy (n = 56)	France (n = 25)	Netherlands (n = 21)	Sweden (n = 36)	All others (n = 40)
ICD implantation	63 (35.4 %)	21 (37.5 %)	6 (24 %)	9 (42.9 %)	11 (30.6 %)	16 (40 %)
Pacemaker implantation	13 (7.3 %)	3 (5.4 %)	0	2 (9.5 %)	5 (13.9 %)	3 (7.5 %)
No implanted devices	95 (53.4 %)	27 (48.2 %)	17 (68 %)	11 (52.4 %)	22 (61.1 %)	18 (45 %)
Myectomy	18 (10.3 %)	7 (12.5 %)	2 (8.3 %)	2 (9.5 %)	5 (13.9 %)	2 (5.3 %)
AFib related procedures	23 (13.1 %)	4 (7.1 %)	7 (29.2 %)	3 (14.3 %)	5 (13.9 %)	4 (10.5 %)
Other HCM-related	20 (11.4 %)	6 (10.7 %)	2 (8.3 %)	3 (14.3 %)	5 (13.9 %)	4 (10.5 %)
No HCM-related surgeries	124 (70.9 %)	43 (76.8 %)	13 (54.2 %)	15 (71.4 %)	24 (66.7 %)	29 (76.3 %)

Genetic testing was performed in 166 (78 %, 95 % CI 72–83, 213 respondents), yet only 41 (68 %, 95 % CI 60–71) of those with a positive genetic result reported family screening, despite high levels of trust in their physicians (279, 83 %, 95 % CI 76–89).

### 2.5 Psychological burden of the disease

**Fig. 5:**
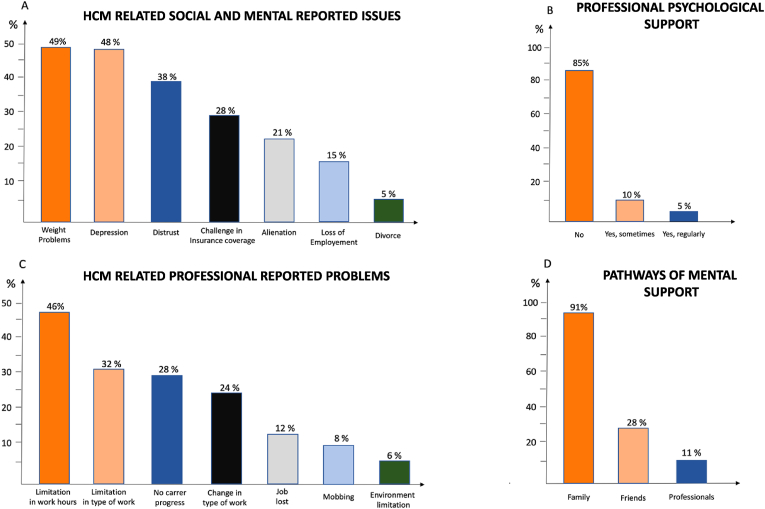
Psychological impact and pathways to seek mental support of the participants.

The psychological burden of HCM emerged as a major concern in the survey, with 138 (41 %, 95 % CI 35–48) of patients expressing fear of disease progression and 86 (82 %, 95 % CI 75–86, among 104 respondents) experiencing anxiety about transmitting the condition to their children. The most common concerns reported were weight problems in 71 (49 %, 95 % CI 40–56) and depression in 70 (48 %, 95 % CI 41–56) participants (Fig. 5). Twenty-two (15 %, 95 % CI 9–19) patients reporting having lost their job because of HCM. Moreover, 46 (46 %, 95 % CI 39–51) reported a limitation in working hours as well as limitation in the kind of work performed (32, 33 %, 95 % CI 25–37), due to the disease (Fig. 5). Family planning decisions were impacted in 29 % (95 % CI 20–32) of respondents, and 34 % (95 % CI 29–39)reconsidered their career paths due to uncertainty regarding the disease's progression.

Despite a significant psychological burden access to mental health support was limited, as only 15 % (95 % CI 10–21) of patients regularly consulted a psychologist, while 22 % (95 % CI 18–26) required medications for anxiety or depression related to their cardiac condition. Most of the support concerning HCM related consequences was provided by family members (91, 91 %, 95 % CI 86–95) (Fig. 5).

## 3 Discussion

This survey represents one of the largest patient-reported datasets on HCM, covering demographics, disease burden, quality of care, psychological well-being, and healthcare access [7,9]. This study aimed to identify the gaps in HCM care and target opportunities for improving patient-centered management strategies. Understanding how patients perceive their disease and its impact on daily life – with a patient-patient interaction - is crucial for developing more effective healthcare policies, optimizing access to specialized care, and ensuring psychological support for those affected by 10.13039/501100014603HCM.

The participants spanned over 18 countries, with at least 4 with more than 50 participants. Interestingly, a diverse geographical distribution was present. The majority of participants (56 %) resided in in small towns/rural or remote areas, translating into a variable access to specialized care. Moreover, almost one third of the participants had to travel over one day to receive specialized treatment, potentially highlighting the need for expanded telemedicine services, regional referral networks, and travel support programs to mitigate geographical barriers to care.

Despite high educational attainment, with 96 % of respondents completing secondary or higher education and 39 % holding a university degree, many patients struggled to maintain stable employment due to HCM-related limitations. 48 % of respondents reported an average or above average impact on their professional life, with 12 % experiencing job loss and 47 % report inability to work the number of desired hours and 24 % had to change the type of work.

Notably, women were more likely to experience work-related disruptions than men (35 % vs. 22 %. 38 % of women vs. only 9 % of men declare that they cannot work the number of hours they desire (p < 0.05). Furthermore, respondents encountered challenges in obtaining workplace accommodations, suggesting insufficient employer awareness and support for individuals with chronic cardiac conditions. These findings reinforce the need for occupational health initiatives, including flexible work policies, employer education programs, and legal protections to ensure that individuals with HCM can maintain meaningful employment without compromising their health, as recently highlighted [10].

HCM symptoms significantly impacted daily life and physical activity. At the time of diagnosis, 42 % of patients experienced shortness of breath and 40 % fatigue. The relative weight of the perceived impact of these symptoms was significant (Fig. 3). Over time, 68 % of respondents continued to report persistent symptoms, with fatigue (45 %) and dyspnea (44 %) remaining the most limiting factors. Notably, symptom trajectory was highly variable, with 42 % experiencing worsening symptoms, 29 % reporting stable symptoms, and 26 % perceiving an improvement, potentially due to optimized treatment strategies or lifestyle adjustments. Physical activity was also significantly affected, with 54 % of patients reducing their activity levels post-diagnosis. At the time of diagnosis, 14 % engaged in competitive sports, 35 % in regular exercise, and 42 % in light recreational activity. However, following diagnosis, the proportion of patients engaged in low to moderate activities increased significantly (87 % vs 50 %, p < 0.01). Concomitantly, one of the major psychological concern was related to weight gain, probably partly promoted by sedentarism. This aspect is outmost importance in HCM, given the established association between obesity and outcome in these patients [11]. Today, a growing body of evidence supports the benefits and safety of different forms of exercise in HCM [12,13]. However, despite adoption of specific recommendation about tailored exercise prescription in most recent guidelines [3], real-world data still point towards the need for a broader activity of personalized exercise counseling and structured rehabilitation programs in these patients, since most of them still declares to be limited by fear.

Interestingly, genetic testing was performed in 77 % of respondents, yet only 68 % of those with a positive genetic result pursued family screening, indicating potential gaps in familial risk assessment and genetic counseling. These findings highlight the need for standardized referral pathways, better implementation of genetic screening programs, and improved patient education regarding potential benefits of the genetic assessment for long-term disease management.

Despite evidences that HCM can be significantly associated with the risk of incident mental disorders, particularly within 1 year after HCM diagnosis [14], there are few reports describing the psychological burden of this disease. Almost half of the respondents (49 %) claimed to have experienced depression because of HCM. Family planning decisions were impacted in 29 % of respondents, and 34 % reconsidered their career paths due to uncertainty regarding the disease's progression. However, despite the significant psychological burden, access to mental health support was limited, as only 15 % of patients regularly consulted a psychologist, while most of the help was from family members and friends. These findings underscore the urgent need for integrated mental health support, structured counseling programs, and specifically peer-support networks, such as patients association or patients discussion groups to help navigate the emotional challenges associated with the disease.

## 4 Limitations

While the HCM Patient Survey 2025 provides valuable insights into the real-world experiences of individuals living with hypertrophic cardiomyopathy (HCM) across Europe, several limitations must be acknowledged. Firstly, selection bias may have influenced the results, as participation was voluntary and conducted online, potentially favoring patients who are more engaged in digital health platforms, advocacy groups, or healthcare networks. This may have underrepresented individuals with lower digital literacy, limited internet access, or less interaction with specialized cardiology centers. Secondly, the self-reported nature of the data introduces the possibility of recall bias. Symptoms, past diagnoses, treatment history, and psychological burden were reported based on patient perception rather than objective clinical assessments, which may lead to variability in accuracy and interpretation. Thirdly, while the survey included participants from 18 European countries, the distribution of responses was not uniform across regions. Countries with higher participation rates may have disproportionately influenced the findings, while low-response countries may not be fully representative of their respective populations. Additionally, healthcare systems, access to genetic testing, and treatment availability vary significantly across Europe, which may limit the generalizability of some findings.

Another important limitation is the lack of clinical correlation with patient-reported outcomes. Although the survey provides rich qualitative and quantitative data, it does not include clinical parameters such as imaging findings, genetic test results, or biomarker assessments, which would have strengthened the correlation between patient-reported burden and disease severity. Lastly, psychological distress and quality of life measures were not assessed using validated clinical scales, potentially limiting the ability to compare these results with formal psychosocial assessments used in clinical research. Future studies should integrate standardized psychological and health-related quality-of-life tools to provide a more comprehensive evaluation of the emotional burden of HCM. Despite these limitations, this study remains one of the most extensive patient-driven surveys on HCM, highlighting critical gaps in healthcare access, disease management, and psychosocial support.

## 5 Conclusions

The HCM Patient Survey 2025 provides insights into the real-world experiences of individuals living with HCM across Europe. The findings highlight critical gaps in healthcare access, symptom management, employment support, and mental health services, underscoring the need for policy-driven changes to optimize patient care. These findings provide a valuable foundation for further research, policy discussions, and advocacy efforts aimed at improving patient-centered care in HCM.

## CRediT authorship contribution statement

**Emil Tsenov:** Investigation, Funding acquisition, Data curation, Conceptualization. **Jolanda Van der Velden:** Writing – review & editing, Validation, Supervision, Formal analysis. **Matteo Pinciroli:** Writing – review & editing, Investigation, Conceptualization. **Maurizio Pieroni:** Writing – review & editing, Conceptualization. **Franco Cecchi:** Writing – review & editing, Formal analysis, Conceptualization. **Iacopo Olivotto:** Writing – review & editing, Data curation, Conceptualization. **Niccolò Maurizi:** Writing – original draft, Validation, Methodology, Formal analysis, Data curation, Conceptualization.

## Disclosures

Emil Tsenov has received fees (honoraria or consulting) from Cytokinetics. Dr. I. Olivotto has received grants from Bristol Myers Squibb, Cytokinetics, Amicus, Genzyme, Shire, Bayer, Boston Scientific, Menarini International, and fees (honoraria or consulting) from Bristol Myers Squibb, Cytokinetics, Amicus, Genzyme, Shire, Boston Scientific, and served or currently serving as PI on EXPLORER‐HCM, MAVA‐LTE, REDWOOD‐HCM, REDWOOD‐OLE, and SEQUOIA‐ HCM trial. Matteo Pinciroli has received fees (honoraria or consulting) from Bristol Myers Squibb. Dr. N Maurizi has received grants from Bristol Myers Squibb, Amicus and Foundation CVCL and fees (honoraria or consulting) from Bristol Myers Squibb and Academic CME.

## Data availability

Data would be available upon reasonable request.

## Funding

An unrestricted grant was provided by Bristol Myers Squibb, Tenaya Therapeutics and Edgewise Therapeutics. Sponsors were not involved in content creation, survey diffusion, promotion or analysis.

## Declaration of competing interest

The authors report no conflict of interest regarding the topic discussed in the present manuscript.
